# MMP-2 associated imbalance of VEGF/Endostatin is linked to suppression of the PI3K/AKT/HIF-1α pathway in steroid-induced osteonecrosis of femoral head

**DOI:** 10.1371/journal.pone.0346880

**Published:** 2026-04-17

**Authors:** Guangyang Zhang, Zhaopu Jing, Jialin Liang, Leifeng Lv, Xiaoqian Dang, Qichun Song

**Affiliations:** Department of Sports Medicine, Orthopedic Center, the Second affiliated hospital of Xi’an Jiaotong University, Xi’an, Shaanxi, China; The Affiliated Changzhou No 2 People’s Hospital of Nanjing Medical University, CHINA

## Abstract

**Objective:**

To investigate the role of matrix metalloproteinase-2 (MMP-2) and the imbalance between vascular endothelial growth factor (VEGF) and Endostatin in the pathogenesis of steroid-induced osteonecrosis of femoral head (SONFH).

**Methods:**

Clinical samples and animal models were combined to systematically analyze the impact of VEGF/Endostatin imbalance on angiogenesis and osteogenesis. Clinical samples from SONFH patients and femoral neck fracture controls were analyzed for VEGF, Endostatin and MMP-2 expression. The SONFH model in C57BL/6J mice was established to evaluate the effects of an imbalanced VEGF/Endostatin axis on angiogenesis and osteogenesis, assessed by micro-CT based vascular imaging, calcein labeling, and bone density analysis. MMP-2 intervention experiments were conducted to explore its role in the VEGF/Endostatin ratio.

**Results:**

Clinical data revealed significantly lower VEGF/Endostatin ratio (P < 0.001) and higher MMP-2 levels (P < 0.05) in necrotic regions of SONFH patients compared to the controls. In animal models, decreased VEGF/Endostatin ratio caused by Endostatin supplementation could further suppress angiogenesis and osteogenesis (P < 0.05). Mechanistically, MMP‑2 disrupts the VEGF/Endostatin balance by decreasing VEGF expression (P < 0.05) and increasing Endostatin levels (P < 0.05), which is associated with the suppression of PI3K/AKT/HIF‑1α pathway.

**Conclusions:**

Our findings reveal that the imbalance of VEGF/Endostatin is a key driver of SONFH through impaired angiogenesis and osteogenesis. We further demonstrate that MMP‑2 contributes to this imbalance, likely linked to suppression of the PI3K/AKT/HIF‑1α pathway. These results support the VEGF/Endostatin ratio as a potential diagnostic biomarker and suggest that targeting MMP‑2 or Endostatin may represent promising therapeutic strategies.

## 1. Introduction

Osteonecrosis of the femoral head (ONFH) is a common disabling orthopedic disorder characterized by the interruption of blood flow to the femoral head, finally resulting in necrosis of bone cells and marrow elements. This pathophysiological process eventually leads to femoral head collapse, joint dysfunction, and the need for total hip arthroplasty [1]. Among the various etiologies of ONFH, steroid-induced osteonecrosis of the femoral head (SONFH) represents one of the most prevalent forms [2]. Since the first clinical report in 1953 linking corticosteroid therapy with femoral head necrosis, the incidence of SONFH has significantly increased, largely due to the extensive use of glucocorticoids in managing autoimmune diseases, transplantation, and severe infections [3]. Although the mechanisms of SONFH are multifactorial, accumulating evidence highlights that impaired angiogenesis during bone remodeling is a critical pathological feature that drives disease progression [4,5]. During the early stages of osteonecrosis, ischemia and hypoxia damage osteocytes and stromal components. If timely neovascularization and osteogenesis occur, necrotic tissue may be replaced and remodeled. However, if angiogenic repair fails, progressive bone deterioration and structural collapse ensue. Therefore, elucidating the regulation of angiogenesis in SONFH is essential for understanding its pathogenesis and developing novel therapeutic strategies.

Vascular endothelial growth factor (VEGF) plays a central role in the initiation and maintenance of angiogenesis. As a pro-angiogenic factor, VEGF promotes endothelial cell proliferation, migration, and new vessel formation [6]. Reduced VEGF expression has been associated with impaired blood supply and delayed bone repair in ONFH. Several experimental studies have shown that VEGF delivery or gene therapy can effectively improve neovascularization and accelerate bone regeneration in osteonecrotic lesions [7]. In contrast, Endostatin, an endogenous angiogenesis inhibitor derived from collagen XVIII, antagonizes VEGF signaling by inhibiting endothelial proliferation and promoting vessel regression [8]. While Endostatin has been extensively studied in tumor biology, its role in SONFH remains insufficiently defined and potentially detrimental. Our previous work in a rabbit SONFH model demonstrated that exogenous Endostatin administration significantly exacerbates bone necrosis and reduces neovascular formation [9], suggesting that anti-angiogenic stress may play a negative role in this context.

The ratio of VEGF to Endostatin expression may thus represent a valuable index reflecting the balance between pro- and anti-angiogenic forces within various diseases. For example, operable non-small cell lung cancer patients have been shown to exhibit a higher VEGF/Endostatin ratio than healthy controls, and this imbalance is associated with poorer prognosis [10]. Additionally, it was revealed that the dynamic changes of VEGF and Endostatin illustrate a shifting angiogenic balance that contributes to hepatocellular carcinoma progression and may hold diagnostic and prognostic value [11]. Furthermore, Ai Jing et al. reported that restoring the VEGF/Endostatin balance by elevating Endostatin levels effectively ameliorated pathological retinal neovascularization [12]. However, the dynamic regulation of this ratio and its pathological consequences in SONFH have yet to be fully clarified. Notably, matrix metalloproteinases (MMPs), particularly MMP-2, are implicated in extracellular matrix remodeling and may indirectly affect the bioavailability and activity of angiogenic factors. MMP-2 is known to degrade extracellular matrix (ECM) components, modulate cytokine gradients, and potentially alter VEGF storage or release, thereby influencing angiogenic potential [13]. Previous research found that MMP-2, VEGF and Endostatin all played critical roles in the healing of NSAID induced small intestinal ulcers in rats [14]. Additionally, Sara A et al. demonstrated that the expression of VEGF, Endostatin, and MMP-2 undergone a complex and tightly coordinated regulation in response to both acute and chronic exercise training in mice [15]. However, the role of MMP-2 in affecting VEGF and Endostatin expression during SONFH remains unexplored. Moreover, identifying early molecular changes in SONFH is critical for timely diagnosis and intervention. While magnetic resonance imaging remains the clinical gold standard for detecting early stage SONFH, it lacks molecular specificity. Therefore, investigating the potential of VEGF/Endostatin ratio as a serum or tissue biomarker may provide a novel avenue for early detection or risk stratification.

In this study, we combined clinical sample analysis with a steroid-induced mouse model of femoral head to investigate the expression pattern and functional implications of the VEGF/Endostatin axis. We further explored the involvement of MMP-2 in disturbing this balance and assessed its impact on angiogenesis and osteogenesis. Through this integrative approach, we aim to clarify the molecular mechanisms underlying impaired vascular remodeling in SONFH and propose novel targets for early diagnosis and therapeutic modulation.

## 2. Materials and methods

### 2.1 Clinical samples

From January 1, 2023 to December 31, 2023, patients diagnosed with SONFH (ARCO stage III–IV) were enrolled as the experimental group, while age, sex, and BMI matched patients undergoing total hip arthroplasty for femoral neck fracture served as the control group with approval from the Ethics Committee of the Second Affiliated Hospital of Xi’an Jiaotong University (Approval No. 2022194). Patients were eligible for inclusion when they met all of the following criteria: age 18–65 years; a clinical and radiological diagnosis of steroid-induced osteonecrosis of the femoral head according to accepted diagnostic criteria; ARCO stage III–IV disease at the time of enrollment; no prior surgical intervention on the affected hip; and the ability to provide written informed consent and comply with study procedures. Patients were excluded if any of the following applied: osteonecrosis due to trauma, alcohol abuse, metabolic bone disease, hemoglobinopathy, or other non-steroidal causes; prior hip surgery, hip infection, or malignant disease involving the hip; systemic conditions likely to confound outcomes (such as severe hepatic or renal failure, uncontrolled diabetes, or active systemic infection); use of investigational drugs within three months prior to enrollment; or inability to provide informed consent or comply with follow-up. All procedures were conducted in accordance with the Declaration of Helsinki. All participants provided written permission for publication, and informed consent was collected from each individual. The femoral head specimens were collected intraoperatively, fixed in 4% paraformaldehyde for 48 hours, decalcified in 10% EDTA solution for four weeks, dehydrated through graded ethanol, and embedded in paraffin for subsequent histopathological analysis.

### 2.2 Animal models

The study was approved by the Biomedical Ethics Committee of Xi’an Jiaotong University Health Science Center (Approval No. XJTUAE2025−2549). Forty-eight eight-week-old male C57BL/6J mice were used, and the modeling period lasted eight weeks. Mice were housed in standard cages with appropriate bedding, ambient temperature 22 ± 2 °C, 12:12 h light: dark cycle, ad libitum access to food and water, and provided nesting material for enrichment. Mice were monitored daily for health and welfare and weighed weekly. Predefined humane endpoints included ≥20% body weight loss, inability to eat or drink, severe or progressive lameness preventing normal feeding or mobility, severe respiratory distress, or moribund state. Animals meeting humane endpoint criteria were euthanized promptly (≤4 h after detection) by overdose of pentobarbital sodium (100 mg/kg, intraperitoneally; Sigma-Aldrich) with confirmation of death. After a 7-day acclimatization period, the mice were randomly divided into four groups: control, SONFH, SONFH with MMP-2 supplementation (SONFH+MMP-2), and SONFH with Endostatin supplementation (SONFH+Endostatin). The SONFH model was induced by intraperitoneal injection of lipopolysaccharide (20 μg/kg; Sigma‑Aldrich, Missouri, USA) on two consecutive days, followed by daily intramuscular injections of methylprednisolone (100 mg/kg; Sigma‑Aldrich, Missouri, USA) into the gluteus maximus for 14 days, then continued every other day [16]. Mice were simultaneously subjected to elevated feeding to encourage upright posture and rotating cage exercise of 2 h/day to increase activity. Mice in the SONFH+MMP-2 group received subcutaneous injections of recombinant MMP-2 (10 μg/kg; MedChemExpress, NJ, USA) every three days [17,18], while those in the SONFH+Endostatin group were administered daily subcutaneous injections of recombinant Endostatin (2 mg/kg; MedChemExpress, NJ, USA) [9]. The control group received equivalent volumes of physiological saline throughout the experiment. All procedures were conducted by trained personnel. The steroid-induced model did not involve surgical procedures. Animals were handled gently to minimize stress. No routine peri-procedural analgesia was required, but any animal showing signs consistent with pain or distress received rescue analgesia per veterinary instruction. Scheduled euthanasia by overdose of pentobarbital sodium (100 mg/kg, intraperitoneally; Sigma-Aldrich) at 8 weeks was performed identically and bilateral femoral heads were harvested and frozen in liquid nitrogen for subsequent analysis. No unplanned or spontaneous deaths occurred during the study.

### 2.3 Hematoxylin and eosin, immunofluorescence staining

Mouse femoral head specimens were fixed in 4% paraformaldehyde for 48 hours, followed by decalcification in 10% EDTA solution at 37 °C with gentle agitation. The decalcification solution was refreshed every 3 days until the tissue was fully softened. After routine paraffin embedding, serial sections were prepared for hematoxylin and eosin (HE) staining to observe bone tissue structure.

As for the Immunofluorescence staining, paraffin sections were deparaffinized, subjected to antigen retrieval, and blocked with 5% goat serum. Sections were then incubated overnight at 4 °C with primary antibodies against VEGF and Endostatin. After washing, sections were incubated for 1 hour at room temperature in the dark with FITC- or Cy3-conjugated secondary antibodies, followed by nuclear counterstaining with DAPI. The slides were mounted and imaged using a fluorescence microscope.

### 2.4 Micro-computed tomography and micro-computed tomography–based angiography

Following euthanasia, femoral heads were harvested from C57BL/6J mice, fixed in 4% paraformaldehyde for 48 hours, and then immersed in 75% ethanol for micro-computed tomography (micro-CT) scanning. The scanning was performed using a Bruker Skyscan system under the following parameters: 70 kV voltage, 200 μA current, 10 μm resolution, 525 ms exposure time, and 180° rotation angle. A calibration phantom was scanned simultaneously for standardization. The raw images were reconstructed using NRecon software (version 1.7.4.2, Bruker), and the following parameters were analyzed using CT Analyser software (version 1.18.8.0, Bruker): bone mineral density (BMD), trabecular number (Tb.N), trabecular thickness (Tb.Th), trabecular separation (Tb.Sp), bone volume (BV), and the bone surface–to–bone volume ratio (BS/BV).

For vascular imaging, mice were anesthetized and perfused via the left ventricle with heparinized saline followed by 4% paraformaldehyde, and subsequently with Microfil contrast agent at a flow rate of 3 mL/min. The aorta was ligated, and the specimens were kept at 4 °C overnight. The femurs were then harvested, fixed in 4% paraformaldehyde for 24 hours, decalcified in 10% EDTA, and scanned using Micro-CT. Scanning parameters were set as follows: 70 kV voltage, 200 μA current, 6.5 μm resolution, 525 ms exposure time, and 180° rotation angle. Calibration with a phantom was performed concurrently. The reconstructed images were used to analyze vascular volume fraction using the same software and methodology.

### 2.5 Calcein staining

Dynamic bone formation in the femoral head was evaluated using Calcein staining. In the sixth week of modeling, mice received an intraperitoneal injection of Calcein (10 mg/kg, dissolved in 2% NaHCO₃ solution; MedChemExpress, NJ, USA), followed by a second injection 6 days later. After euthanasia, femoral heads were harvested, fixed in 4% paraformaldehyde for 48 hours, and dehydrated through a graded ethanol series. Specimens were then embedded in methyl methacrylate, and continuous sections were prepared. Hard tissue microtome was used for precise sectioning, and calcein fluorescence signals were acquired using a laser scanning confocal microscope. The mineral apposition rate (MAR) was quantitatively analyzed to assess the rate of new bone formation.

### 2.6 Quantitative real-time polymerase chain reaction

Total RNA was isolated from femoral head samples using TRIzol reagent (Thermo Fisher Scientific, MA, USA) following the manufacturer’s instructions. The concentration and purity of RNA were determined with a NanoDrop spectrophotometer (Invitrogen, USA). Subsequently, complementary DNA (cDNA) was synthesized using the PrimeScript RT Master Mix (Takara, Japan) according to the standard protocol. Quantitative real-time PCR (RT-qPCR) was carried out using SYBR Select Master Mix (Vazyme, Nanjing, China) on a CFX96 real-time PCR detection system (Bio-Rad, Hercules, USA). GAPDH was used as the internal reference gene, and relative mRNA expression levels were calculated using the 2^−ΔΔCt^ method. Primer sequences are listed in S1 Table.

### 2.7 Western blotting

Proteins were extracted from femoral head tissues using a standard lysis protocol, and total protein concentrations were determined using a BCA Protein Assay Kit (Beyotime, Shanghai, China). Equal amounts of protein were subjected to SDS-PAGE and subsequently transferred onto 0.22 μm PVDF membranes (Millipore, USA). Membranes were blocked with 5% non-fat milk at room temperature for 2 hours and then incubated overnight at 4 °C with primary antibodies (1:1000 dilution). After washing, the membranes were incubated with horseradish peroxidase (HRP)-conjugated secondary antibodies (1:5000 dilution) for 1 hour at room temperature. Protein bands were visualized using an enhanced chemiluminescence detection kit (Millipore, USA), and the grayscale intensity of each band was quantified using ImageJ software. Detailed information on the antibodies used is provided in S2 Table.

### 2.8 Statistical analysis

Statistical analyses were conducted using SPSS 23.0 (IBM) and GraphPad Prism 9.0. Continuous data are expressed as mean ± SD. Comparisons between two groups used independent t-tests, while categorical variables were analyzed with Chi-square test. Multigroup comparisons employed one-way ANOVA (parametric method) based on normality assessment, with post hoc testing where appropriate. Statistical significance was set at P < 0.05.

## 3. Results

### 3.1 The VEGF/Endostatin ratio decreases but MMP-2 increases in SONFH patients

**Fig 1:**
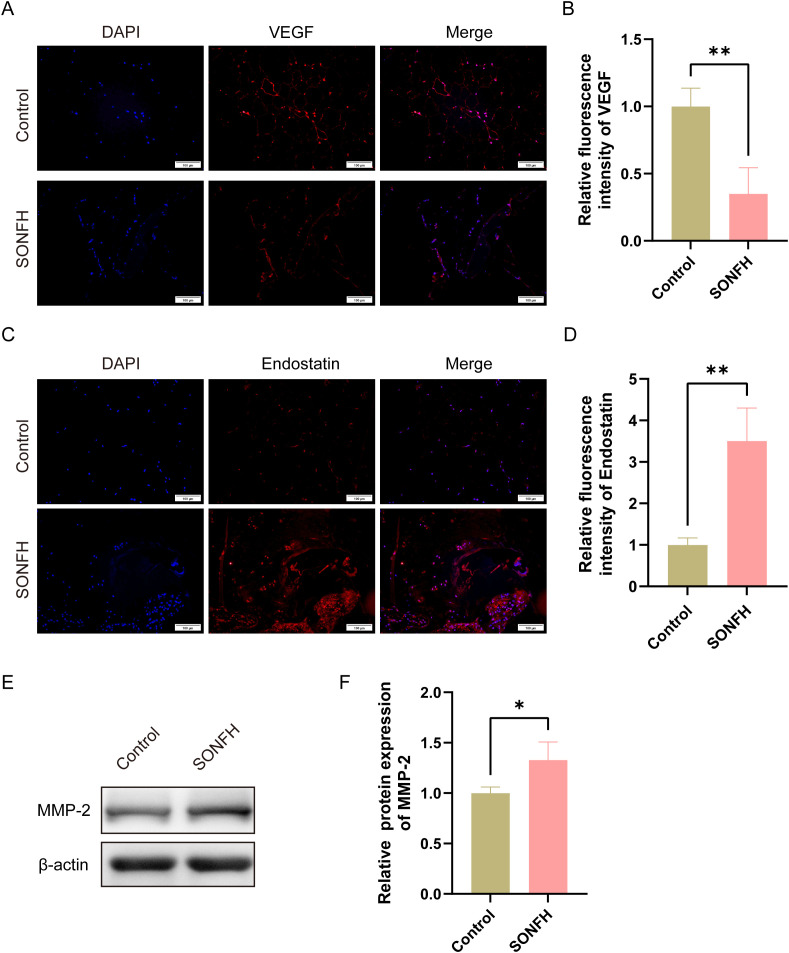
Expression changes of VEGF/Endostatin and MMP-2 in the femoral head of SONFH patients. **(A)** Representative immunofluorescence images showing VEGF expression in the femoral head of control and SONFH groups (scale bar = 100 μm); **(B)** Quantification of VEGF fluorescence intensity in control and SONFH groups; **(C)** Representative immunofluorescence images showing Endostatin expression in the femoral head of control and SONFH groups (scale bar = 100 μm); **(D)** Quantification of Endostatin fluorescence intensity in these two groups; **(E)** Representative Western blotting images of MMP-2 expression in the femoral head of control and SONFH groups; **(F)** Quantification of MMP-2 expression in these two groups. (*P < 0.05, **P < 0.01).

To investigate angiogenic and anti-angiogenic factors in SONFH, femoral head samples were collected from patients with steroid-induced osteonecrosis. Samples from patients undergoing femoral head replacement for femoral neck fractures were collected as controls. Following routine fixation, decalcification, and paraffin embedding, immunofluorescence was performed to assess VEGF and Endostatin expression. As shown in Fig 1A–1D, the SONFH group exhibited a marked reduction in VEGF expression compared to the control group (P < 0.01), alongside a significant increase in Endostatin levels (P < 0.01). Consequently, the VEGF/Endostatin ratio was significantly lower in osteonecrotic femoral heads than in controls (2.991 ± 0.113 vs 0.283 ± 0.098; P < 0.001). Additionally, the level of MMP-2 in the femoral heads was detected by Western blotting method. As shown in Fig 1E and 1F, MMP-2 expression was significantly increased in the SONFH groups compared with the control group (P < 0.05). These findings suggest that the pathological progression of osteonecrosis is closely associated with a disturbed balance of VEGF/Endostatin ratio and MMP-2 expression.

### 3.2 Establishment of a SONFH mouse model and angiogenesis assessment

**Fig 2:**
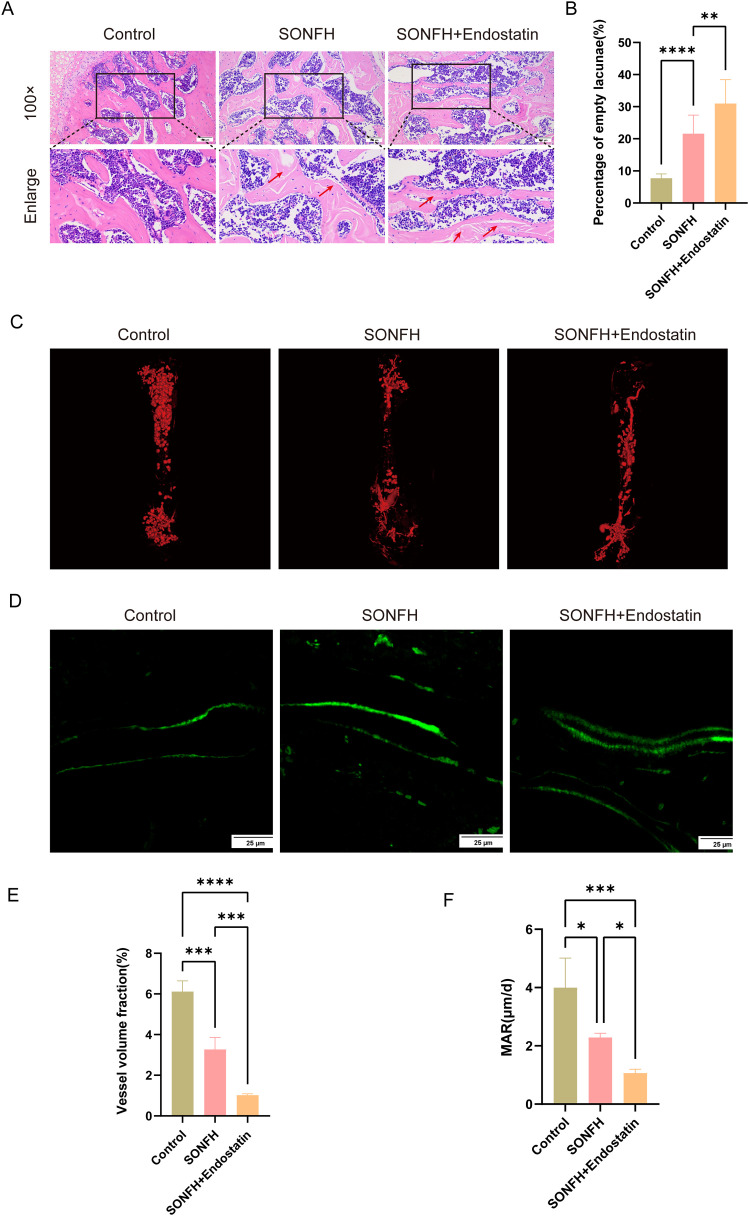
Angiogenic and osteogenic phenotypic changes following Endostatin supplementation in the femoral head of mice. **(A)** Representative HE staining images of the femoral head in control, SONFH, and SONFH+Endostatin groups (scale bar = 100 μm); **(B)** Quantitative analysis of percentage of empty osteocyte from HE staining; **(C)** Representative micro-CT vascular reconstructions of the femoral head in each group; **(D)** Calcein staining images showing mineral apposition in each group (scale bar = 25 μm); **(E)** Quantitative analysis of vascular volume fraction from micro-CT angiography; **(F)** Quantification of mineral apposition rate (MAR) in each group. (*P < 0.05, ***P < 0.001, ****P < 0.0001).

To further explore the mechanisms underlying impaired angiogenesis in osteonecrosis, particularly the imbalance between pro- and anti-angiogenic factors, a steroid-induced osteonecrosis model was established in 8-week-old male C57BL/6 mice. Macroscopic observation revealed that femoral heads in all groups maintained smooth surfaces and regular shapes, with no apparent collapse. Histological examination via HE staining (Fig 2A) revealed preserved trabecular architecture, rich hematopoietic marrow, and few adipocytes in the control group. In contrast, SONFH mice demonstrated sparse, irregular, and fractured trabeculae, disrupted marrow hematopoiesis, and increased adipocyte infiltration. Notably, further elevation of anti-angiogenic pressure through Endostatin supplementation aggravated these histopathological alterations, accompanied by more pronounced nuclear pyknosis, karyorrhexis, and the appearance of empty osteocyte lacunae (red arrows). The percentage of empty osteocytes was quantified and is shown in Fig 2B. Compared with the control group, the SONFH group exhibited a significant increase in the percentage of empty osteocytes (P < 0.0001), which was further increased upon Endostatin supplementation (P < 0.01).

Additionally, micro-CT-based angiography was conducted to evaluate vascular numbers and density (Fig 2C). Quantitative analysis revealed a significant reduction in vascular volume fraction in the SONFH group compared to the controls (Fig 2E, P < 0.001). Endostatin supplementation further suppressed vascular formation (P < 0.001), suggesting that a shift toward an anti-angiogenic balance markedly impairs femoral head angiogenesis under pathological conditions.

### 3.3 Altered osteogenic phenotypes following Endostatin supplementation in the femoral head

Calcein staining was then used to assess the effect of Endostatin supplementation on osteogenesis by measuring the mineral apposition rate (MAR). Compared with controls, SONFH mice displayed a narrower inter-label distance and reduced MAR (Fig 2D and 2F; P < 0.05), and Endostatin supplementation further diminished mineralization capacity (P < 0.05), consistent with a further perturbation of the osteogenic microenvironment.

**Fig 3:**
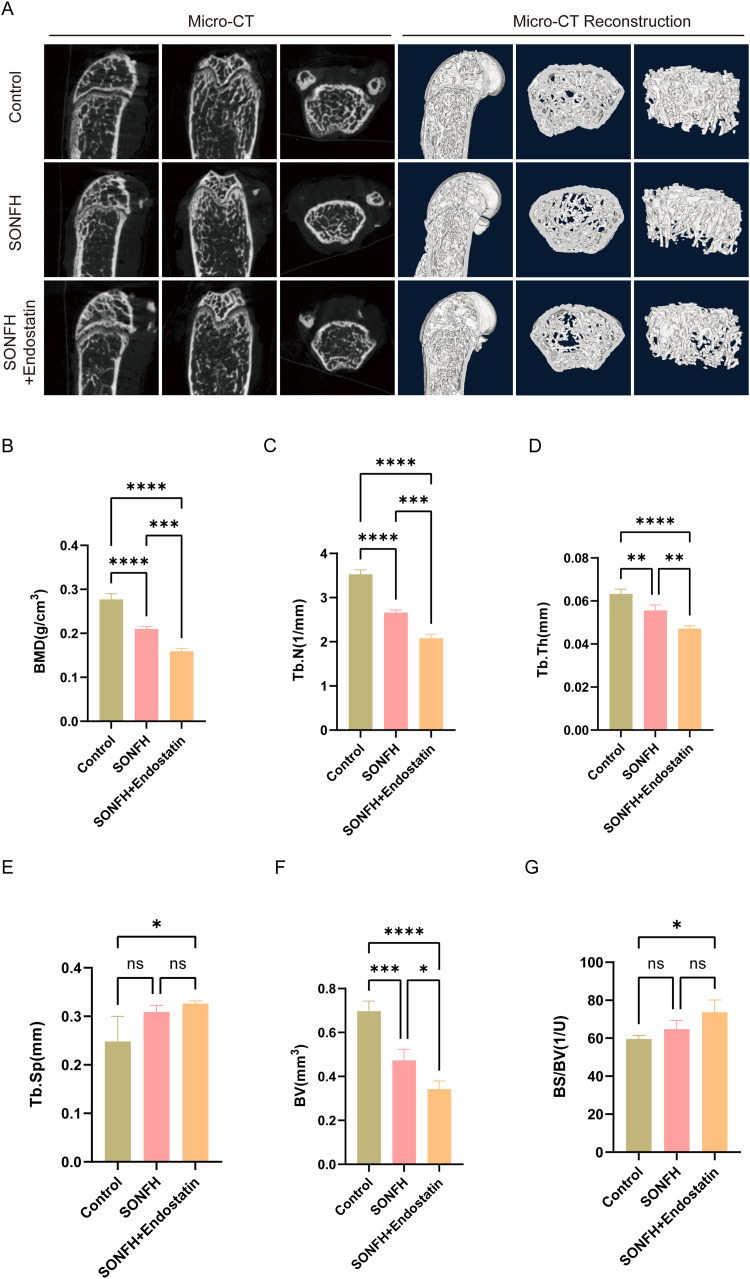
Micro-CT analysis of femoral heads in different experimental groups. **(A)** Representative micro-CT images of femoral heads; **（B）** Quantitative analysis of bone mineral density (BMD); **(C)** Quantification of trabecular number (Tb.N); **(D)** Quantification of trabecular thickness (Tb.Th); **(E)** Quantification of trabecular separation (Tb.Sp); **(F)** Quantification of bone volume (BV); **(G)** Quantification of the bone surface–to–bone volume ratio (BS/BV). (BMD: bone mineral density; Tb.N: trabecular number; Tb.Th: trabecular thickness; Tb.Sp, trabecular separation; BV, bone volume; BS/BV, bone surface–to–bone volume ratio. (*P < 0.01, ***P < 0.001, ****P < 0.0001).

To validate these findings, micro-CT was used to assess bone architecture, including BMD, Tb.N, Tb.Th, Tb.Sp, BV, and BS/BV (Fig 3A). The SONFH group exhibited a marked reduction in BMD relative to controls (Fig 3B; P < 0.0001), which was exacerbated by Endostatin supplementation (P < 0.001). Similarly, Tb.N, Tb.Th, and BV were all significantly decreased following Endostatin supplementation (Fig 3C, 3D and 3F; P < 0.05). In contrast, Tb.Sp did not differ significantly between control and SONFH groups (Fig 3E; P > 0.05); Endostatin supplementation had no effect on Tb.Sp compared with SONFH alone, although Tb.Sp remained higher than in controls (P < 0.05). The BS/BV ratio mirrored the pattern observed for Tb.Sp: Endostatin supplementation increased BS/BV relative to controls (Fig 3G; P < 0.05), but no differences were detected between control versus SONFH or SONFH versus SONFH+Endostatin. Together, these data demonstrate that Endostatin supplementation exacerbates disruption of the osteogenic microenvironment and adversely affects bone remodeling in the femoral head. These osteogenic impairments are consistent with the detrimental effects on angiogenesis observed earlier, highlighting the coupled nature of vascular and bone remodeling processes in SONFH.

### 3.4 MMP-2 decreases VEGF level but enhances Endostatin expression and may contribute to osteonecrosis progression

**Fig 4:**
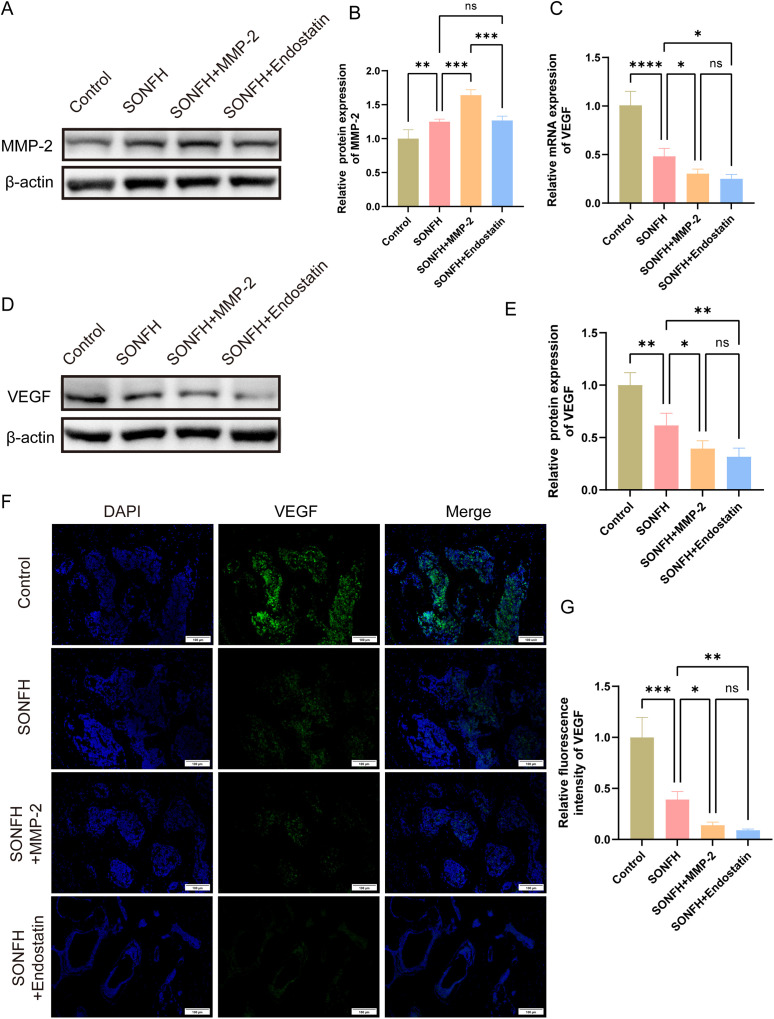
Influence of MMP-2 on VEGF expression. **(A)** Western blotting images showing MMP-2 protein expression; **(B)** Quantification of MMP-2 protein expression; **(C)** Relative mRNA levels of VEGF in the femoral head tissues of each group; **(D)** Western blotting images showing VEGF protein expression; **(E)** Quantification of VEGF protein expression; **(F)** Representative immunofluorescence images showing VEGF expression; **(G)** Quantitative analysis of VEGF immunofluorescence intensity. (*P < 0.05, **P < 0.01, ***P < 0.001).

To elucidate the role of MMP-2 in affecting angiogenic factors during steroid-induced osteonecrosis of the femoral head, mice were randomized into four groups: control, SONFH, SONFH+Endostatin, and SONFH+MMP-2. We first confirmed MMP-2 expression by Western blotting. Compared with controls, MMP-2 protein levels were significantly elevated in the SONFH group (Fig 4A, 4B; P < 0.01). Exogenous MMP-2 supplementation further increased MMP-2 expression in the femoral head (P < 0.001), whereas Endostatin supplementation had no appreciable effect on MMP-2 levels (P > 0.05).

Next, we assessed VEGF expression at both the mRNA and protein levels. RT-qPCR revealed a marked reduction in VEGF mRNA in the SONFH group relative to controls (P < 0.0001). Supplementation with either Endostatin or MMP-2 resulted in additional downregulation of VEGF mRNA (P < 0.05), implicating the role of MMP-2 in VEGF suppression during osteonecrosis. Consistent with these findings, Western blotting and immunofluorescence analyses demonstrated significantly lower VEGF protein in SONFH femoral heads (Fig 4E and 4G; P < 0.01), with further suppression upon Endostatin and MMP-2 supplementation (P < 0.01 and P < 0.05, respectively). No significant difference was observed between the Endostatin and MMP-2 supplementation groups. Collectively, these data indicate that MMP-2, like Endostatin, critically contributes to angiogenic insufficiency in SONFH by downregulating VEGF.

**Fig 5:**
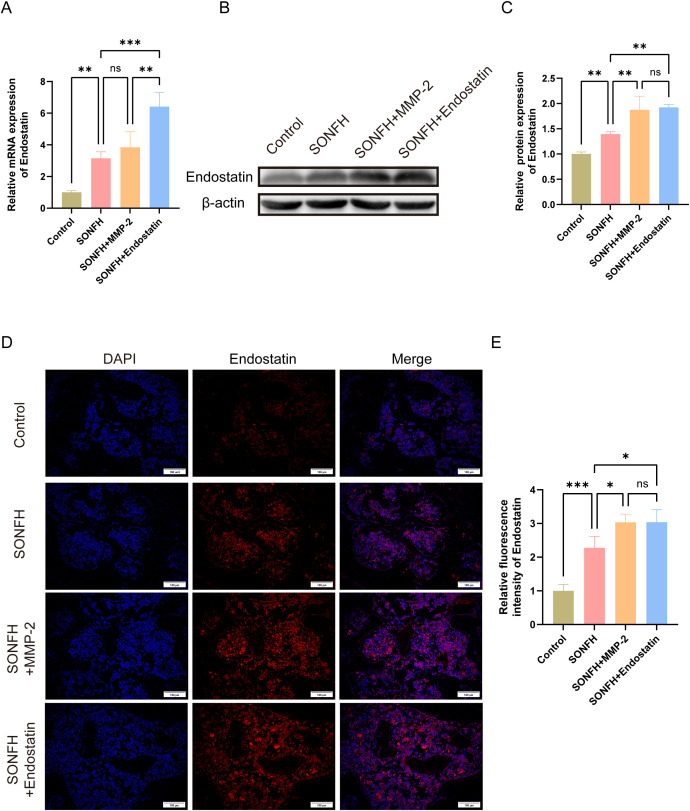
Effect of MMP-2 on Endostatin expression. **(A)** Relative mRNA levels of Endostatin in the femoral head tissues of each group; **(B)** Western blotting images showing Endostatin protein expression; **(C)** Quantification of Endostatin protein levels; **(D)** Representative immunofluorescence images of Endostatin expression; **(E)** Quantitative analysis of Endostatin immunofluorescence intensity. (*P < 0.05, **P < 0.01, ***P < 0.001).

Moreover, to determine whether MMP-2 also influences Endostatin expression, we measured Endostatin levels in all groups by RT-qPCR, Western blotting, and immunofluorescence. Endostatin mRNA was significantly upregulated in the SONFH group versus controls (Fig 5A; P < 0.01), but remained unchanged following MMP-2 supplementation (P > 0.05), suggesting that MMP-2 does not directly regulate Endostatin transcription. Western blotting confirmed increased Endostatin protein in SONFH mice (Fig 5B; P < 0.01). And MMP-2 supplementation modestly elevated Endostatin protein levels (P < 0.01), while exogenous Endostatin supplementation also significantly enhanced endogenous Endostatin levels (P < 0.01), suggesting a potential additive or feedback mechanism. Immunofluorescence mirrored these results, showing that Endostatin expression was higher in the SONFH group compared to controls (Fig 5C; P < 0.001) and was further increased in both MMP-2 and Endostatin intervened mice (P < 0.05). Together, these findings indicate that although MMP-2 does not transcriptionally modulate Endostatin, it may potentiate Endostatin protein accumulation through other mechanisms. Additionally, we calculated the VEGF/Endostatin ratio according to the immunofluorescence results. The control group had a ratio of 1.840 ± 0.597, whereas the SONFH group was significantly lower (0.303 ± 0.024; P < 0.05). Further decreases were observed in the SONFH+MMP-2 (0.054 ± 0.015) and SONFH+Endostatin (0.087 ± 0.023) groups compared with the SONFH group (P < 0.001 and P < 0.01, respectively). No significant difference was found between the SONFH+MMP-2 and SONFH+Endostatin groups (P > 0.05). Taken together, these findings indicate that the detrimental effects of Endostatin supplementation are mediated not by Endostatin alone, but by a shift toward an anti-angiogenic VEGF/Endostatin balance.

### 3.5 MMP-2 is linked to suppression of PI3K/AKT/HIF-1α pathway

**Fig 6:**
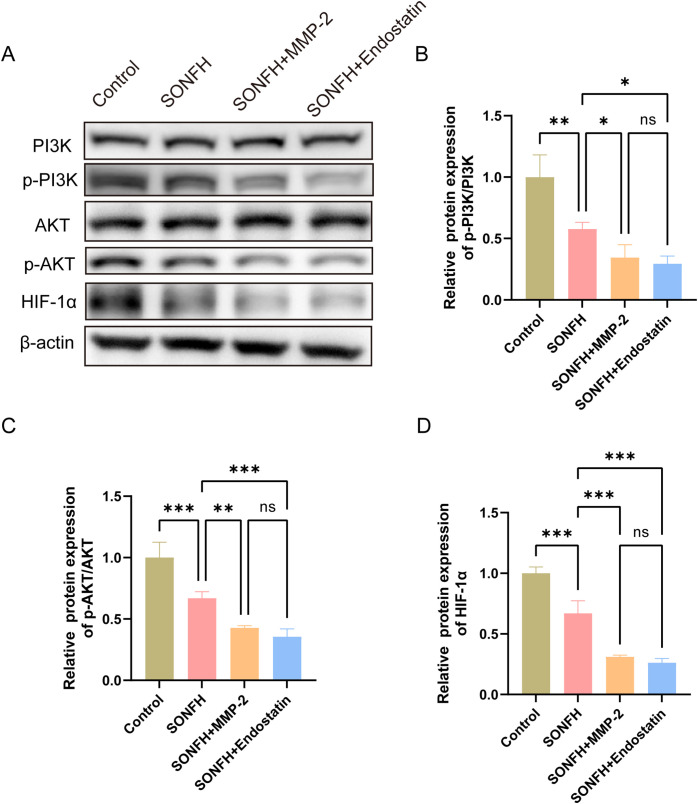
The expression of PI3K/AKT/HIF-1α axis in different experimental groups. **(A)** Western blotting images showing PI3K, AKT and HIF-1α protein expressions; **(B)** Quantification of p-PI3K protein levels; **(C)** Quantification of p-AKT protein levels; **(D)** Quantification of HIF-1α protein levels. (*P < 0.05, **P < 0.01, ***P < 0.001).

As the PI3K/AKT/HIF-1α pathway is essential for both angiogenesis and osteogenesis, we hypothesized that MMP-2 disrupts the VEGF/Endostatin balance by suppressing this signaling pathway. We therefore measured key pathway proteins by Western blotting across all four groups (Fig 6). Phosphorylated PI3K (p-PI3K) was significantly reduced in the SONFH group versus controls (Fig 6A and 6B; P < 0.01), and supplementation with either MMP-2 or Endostatin further decreased p-PI3K levels (P < 0.05), with no difference between the two supplementations. Similarly, both p-AKT and HIF-1α were markedly lower in SONFH mice compared to controls (Fig 6C and 6D; P < 0.001), and supplementation of MMP-2 or Endostatin caused additional reductions in p-AKT and HIF-1α (P < 0.01). The results indicate that glucocorticoid impairs the PI3K/AKT/HIF-1α axis in the femoral head and that both MMP-2 and Endostatin exacerbate this impairment.

## 4. Discussion

Osteonecrosis of the femoral head is characterized by interrupted blood supply to the femoral head, leading to ischemia, bone cell death, structural collapse, and ultimately the need for joint replacement. ONFH primarily affects individuals between 30 and 50 years of age, and it is estimated that 5% to 12% of total hip arthroplasty procedures are necessitated by ONFH [19]. Therefore, understanding the molecular regulation is critical to the development of early diagnostic markers and therapeutic strategies.

VEGF is a central pro-angiogenic factor that promotes endothelial proliferation, migration and vessel formation and is closely linked to osteogenesis. Previous studies report that VEGF augments both angiogenesis and bone formation in diverse repair models [20,21]. Endostatin, a collagen-XVIII derived endogenous angiogenesis inhibitor, counteracts VEGF-mediated endothelial responses and has been shown to suppress angiogenesis in bone healing contexts [22]. The physical exercise was shown to increase skeletal muscle VEGF expression while suppressing Endostatin expression, and a negative correlation was observed between Endostatin levels and capillary density [23]. Moreover, altered VEGF/Endostatin balance contributes to impaired angiogenesis in systemic sclerosis, and both of them can be biomarkers of systemic sclerosis [24,25]. Clinically, imbalances in VEGF and Endostatin expression have been linked to disease progression in melanoma, with stage III patients showing elevated Endostatin and reduced VEGF levels, while stage IV patients exhibited increased levels of both factors [26]. These findings suggest that the VEGF/Endostatin axis may serve not only as a therapeutic target but also as a potential biomarker for disease stage and severity.

Our study demonstrated that the VEGF/Endostatin ratio was significantly reduced in SONFH patients and mice. VEGF expression was downregulated, whereas Endostatin expression was increased. This imbalance suggests a shift toward an anti-angiogenic environment in the femoral head during SONFH progression. Importantly, although Figs 2 and 3 primarily depicts phenotypic outcomes, the subsequent analyses in Figs 4 and 5 demonstrate that Endostatin supplementation alters VEGF/Endostatin dynamics, decreasing VEGF levels while increasing Endostatin, which leads to a reduced VEGF/Endostatin ratio and provides a mechanistic explanation for the observed changes in angiogenesis and osteogenesis. Collectively, these data indicate that dysregulation of the VEGF/Endostatin axis is closely associated with impaired femoral head repair in SONFH.

MMP-2 has been implicated in the regulation of angiogenic factors, but its effects appear context-dependent. It should be noted that MMP-2 is a matrix-associated protease whose biological functions are highly dependent on the extracellular matrix context, which may not be fully recapitulated in simplified in vitro systems [27–29]. Some reports link MMP-2 inhibition to reduced VEGF and impaired angiogenesis, while others show that MMP-2 suppression can increase VEGF availability and promote repair, indicating tissue and disease specific roles [30–34]. These divergent findings underscore the complex regulatory role of MMP-2 in angiogenesis. In our study, elevated MMP-2 levels were observed in clinical samples from SONFH patients. Similarly, in the SONFH animal model, increased MMP-2 was accompanied by decreased VEGF, increased Endostatin, and a reduced VEGF/Endostatin ratio, mirroring the clinical findings. And both MMP-2 and Endostatin correlated with suppression of PI3K/AKT/HIF-1α signaling. Excess MMP-2 activity may cleave basement membrane collagen XVIII, increasing extracellular Endostatin. Although ECM degradation by MMP-2 can release matrix-bound VEGF, proteolytic processing may limit VEGF bioavailability [35]. Activation of the PI3K/AKT/HIF-1α axis is known to upregulate VEGF expression [36]. In this study, increased Endostatin was associated with downregulation of PI3K/AKT/HIF-1α and reduced VEGF. Previous reports have shown that Endostatin can decrease HIF-1α expression [37,38]. Taken together, these results suggest that MMP‑2 affects the VEGF/Endostatin ratio and is associated with the suppression of PI3K/AKT/HIF‑1α pathway. However, the precise molecular links among MMP-2, Endostatin and PI3K/AKT/HIF-1α remain to be defined.

Current therapeutic strategies for early stage SONFH are limited and largely palliative. Our data suggest that restoring a favorable VEGF/Endostatin ratio either by enhancing VEGF, inhibiting Endostatin, or modulating MMP-2 may be a promising approach to improve neovascularization and osteogenesis. The drugs selectively inhibit Endostatin could be developed to promote angiogenic repair in the early stages of SONFH. Moreover, the VEGF/Endostatin ratio itself may serve as a novel biomarker for early diagnosis, treatment monitoring, or prognostic prediction.

Despite these encouraging results, several limitations of this study should be acknowledged. First, although MMP-2 was associated with a disturbed VEGF/Endostatin balance and reduced PI3K/AKT/HIF-1α signaling, causality was not established. To address this, we will integrate ECM-enriched in vitro models together with in vivo MMP-2 knockdown and overexpression approaches to determine whether modulation of MMP-2 directly regulates angiogenic signaling, VEGF/Endostatin balance, and SONFH related phenotypes. Second, it remains unclear to what extent Endostatin mediates the effects of MMP-2. In the subsequent analyses, Endostatin knockdown during MMP-2 overexpression and adding recombinant Endostatin after MMP-2 inhibition will be performed. These experiments will clarify how strongly MMP-2 induced deterioration depends on Endostatin. Another limitation of this study is that the analyses were performed on whole femoral head tissue, which contains multiple cell types and therefore reflects averaged signals rather than cell-type–specific changes. Future studies will employ cell-type–specific approaches, such as laser-capture microdissection, cell sorting, and spatial transcriptomics, to further clarify the underlying mechanisms.

## 5. Conclusion

In summary, our study demonstrates that the VEGF/Endostatin ratio is markedly reduced in SONFH, and that further disruption of this balance exacerbates impairments in both angiogenesis and osteogenesis. Moreover, MMP‑2 affects this ratio and is linked to the suppression of PI3K/AKT/HIF‑1α pathway. These findings deepen our understanding of SONFH pathogenesis and identify new molecular targets for its diagnosis and treatment.

## Supporting information

S1 Table Primers for RT-qPCR. (PDF)

S2 Table Antibodies for WB and IF staining. (PDF)

S1 File Raw images. (PDF)

S2 File The minimal anonymized dataset necessary. (XLSX)
