# Supplementary material for: MMP-2 associated imbalance of VEGF/Endostatin is linked to suppression of the PI3K/AKT/HIF-1α pathway in steroid-induced osteonecrosis of femoral head
Source: PLoS One. 2026 Apr 17;21(4):e0346880. doi: 10.1371/journal.pone.0346880 (PMC13089727; doi:10.1371/journal.pone.0346880)
Supplement: S1 Table — (PDF) [file pone.0346880.s001.pdf]

Table S1 Primers for RT-qPCR

Gene	Forward Primer	Reverse Primer
Mus GAPDH	ATGGGTGTGAACCACGAGA	CAGGGATGATGTTCTGGGCA
Mus VEGF	GCACCCACGACAGAAGGAG	GCATCAGCGGCACACAGGA
Mus Endostatin	GTGACACTGGACCTCAAGGCTT	TTGTCTGAAGGAGGGTCCTGGT

GAPDH, Glyceraldehyde 3-phosphate dehydrogenase; VEGF, Vascular endothelial growth factor.
