# Supplementary material for: MMP-2 associated imbalance of VEGF/Endostatin is linked to suppression of the PI3K/AKT/HIF-1α pathway in steroid-induced osteonecrosis of femoral head
Source: PLoS One. 2026 Apr 17;21(4):e0346880. doi: 10.1371/journal.pone.0346880 (PMC13089727; doi:10.1371/journal.pone.0346880)
Supplement: S2 Table — (PDF) [file pone.0346880.s002.pdf]

Table S2 Antibodies for WB and IF staining

Experiment	Antibody Name	Supplier	Code
WB	β-actin	Proteintech	66009-1-Ig
	Endostatin	LSBio	LS-C806230
	VEGF	Proteintech	19003-1-AP
	MMP-2	Abcam	ab92536
	PI3K	CST	4257
	p-PI3K	CST	17366
	AKT	CST	9272
	p-AKT	CST	4060
	HIF-1α	CST	14179
IF	VEGF	Proteintech	19003-1-AP
	Endostatin	LSBio	LS-C806230

VEGF, vascular endothelial growth factor.
