# Supplementary figures and images for: MMP-2 associated imbalance of VEGF/Endostatin is linked to suppression of the PI3K/AKT/HIF-1α pathway in steroid-induced osteonecrosis of femoral head
Source: PLoS One. 2026 Apr 17;21(4):e0346880. doi: 10.1371/journal.pone.0346880 (PMC13089727; doi:10.1371/journal.pone.0346880)

# MMP-2

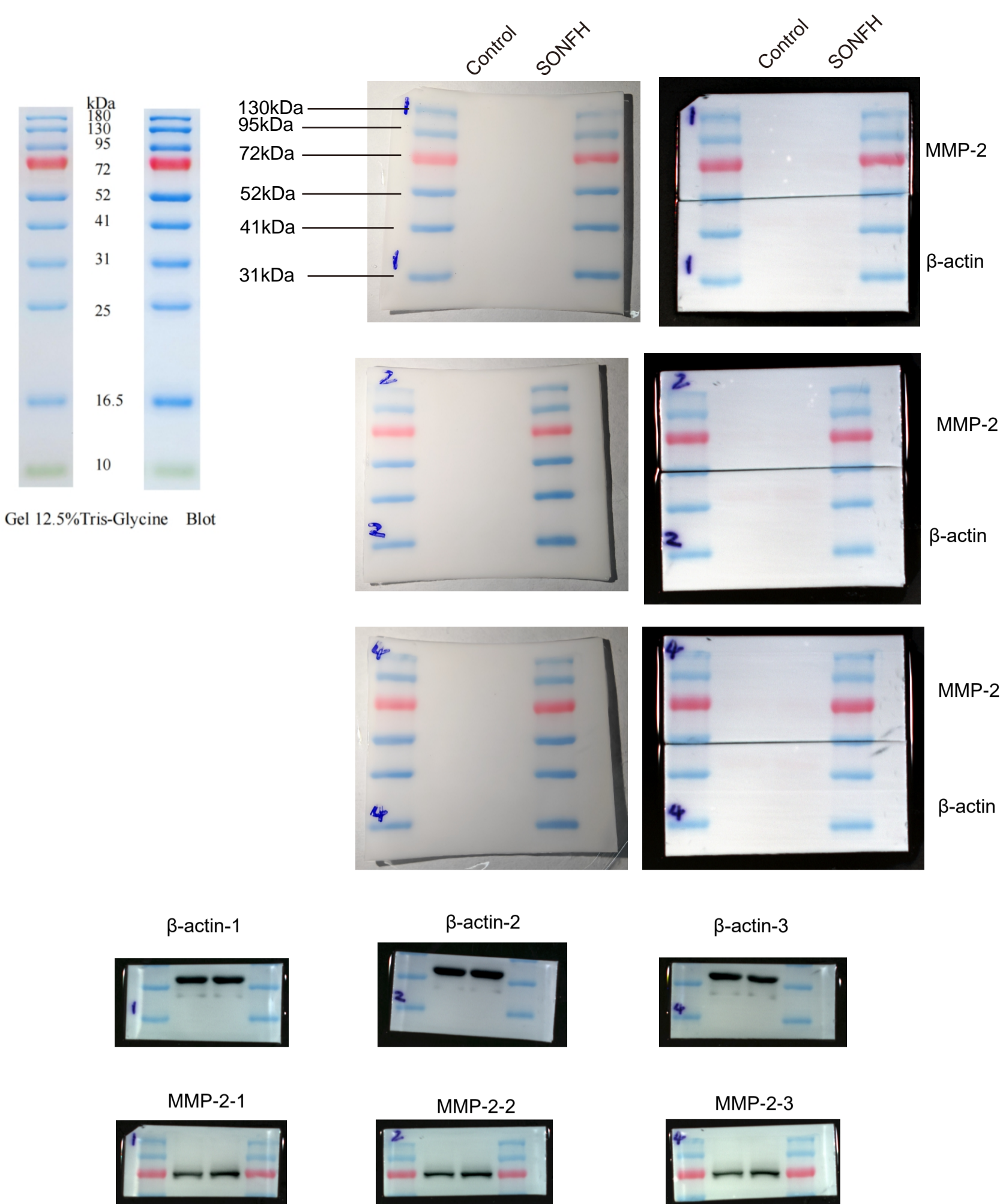

# VEGF/Endostatin/MMP-2

## VEGF/MMP-2

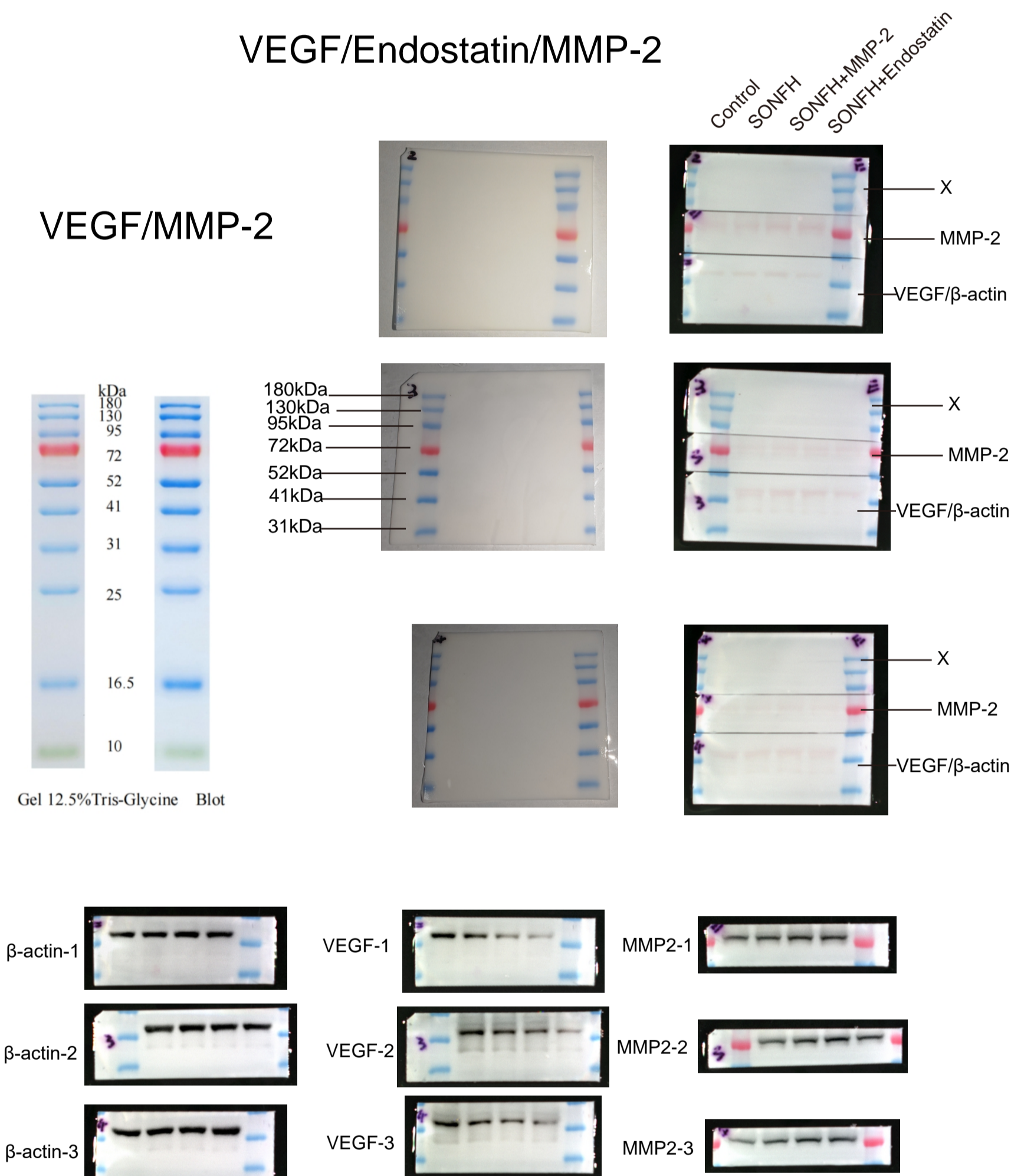

## Endostatin

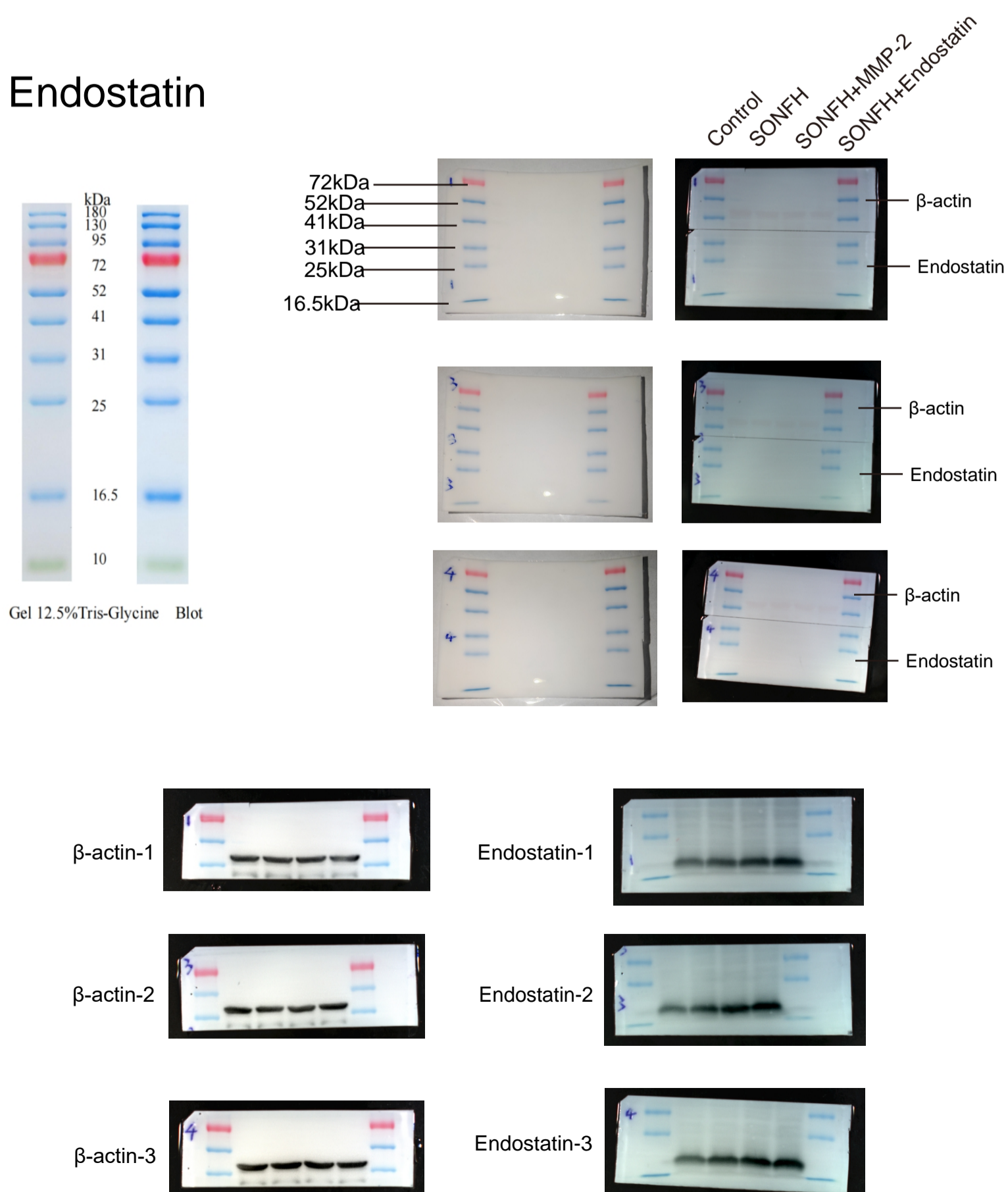

P13K/AKT/HIF-1

p-PI3K/PI3K

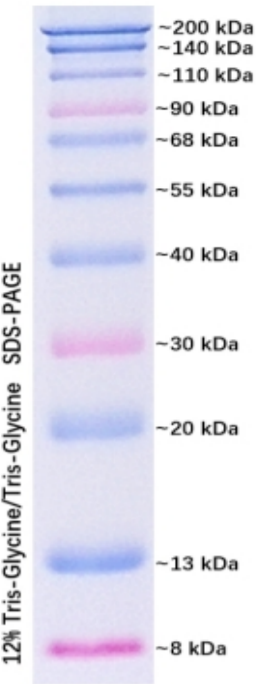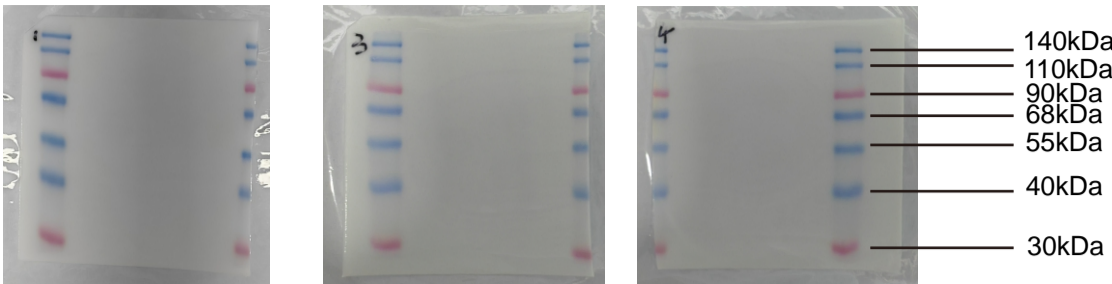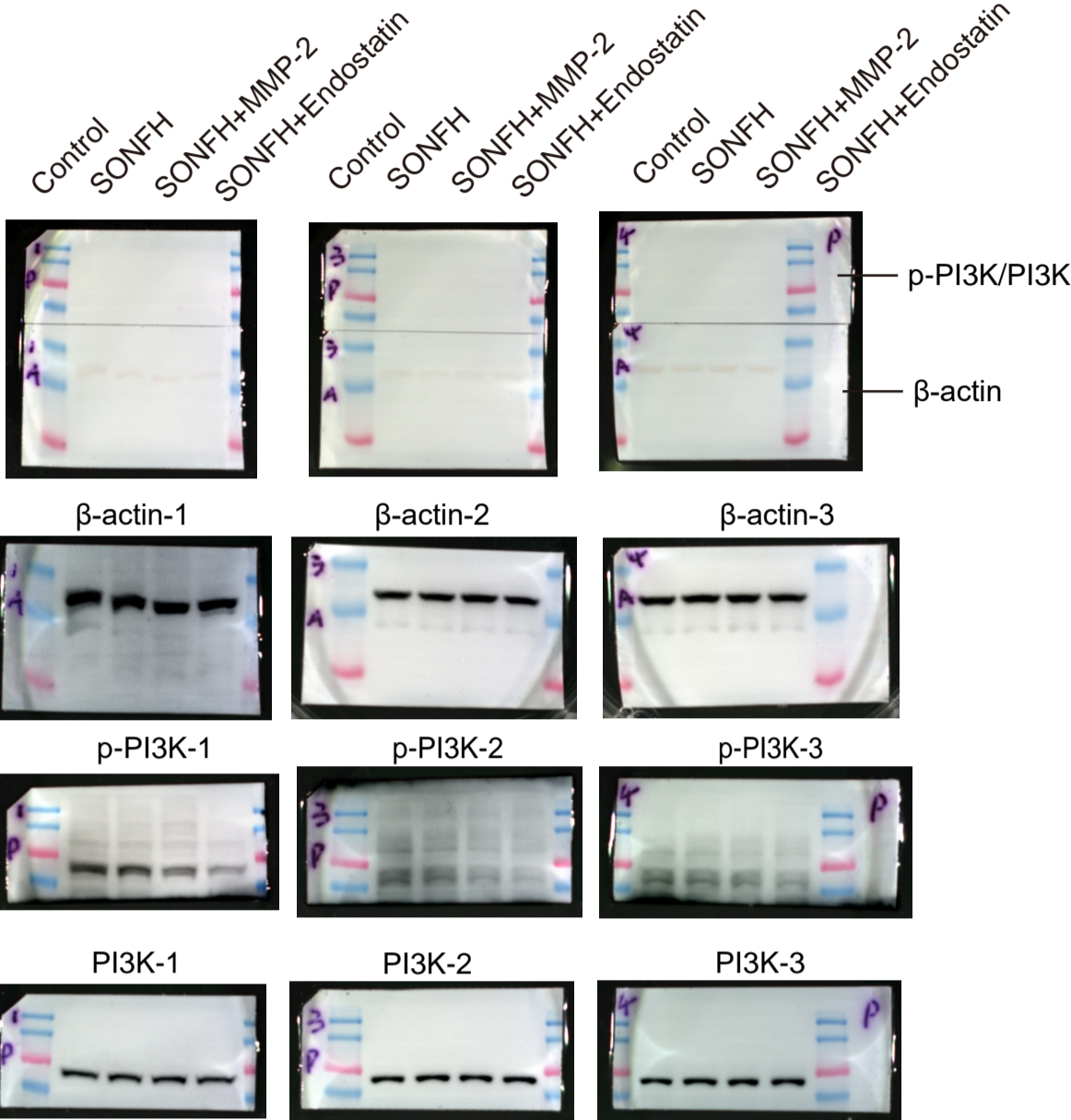

p-AKT/AKT/HIF-1α

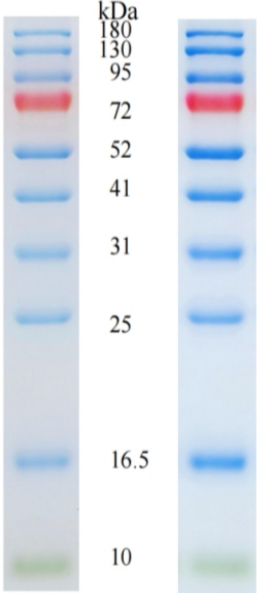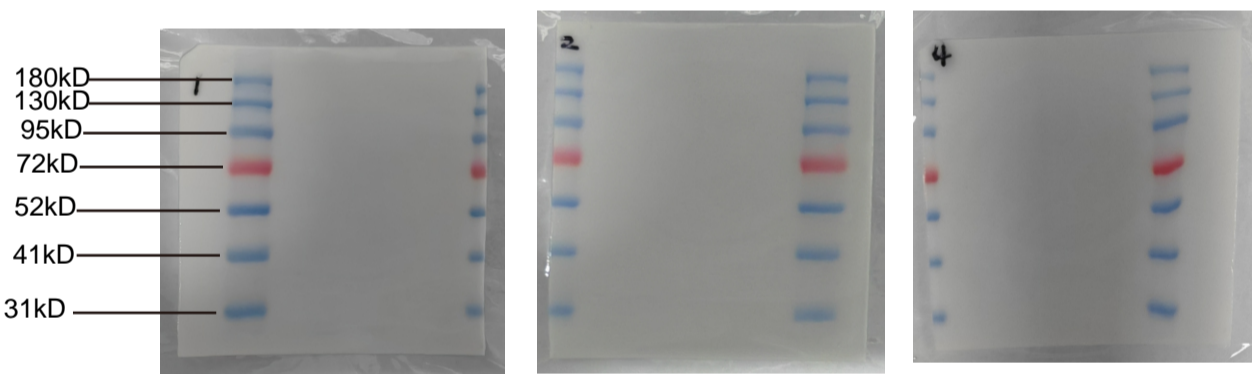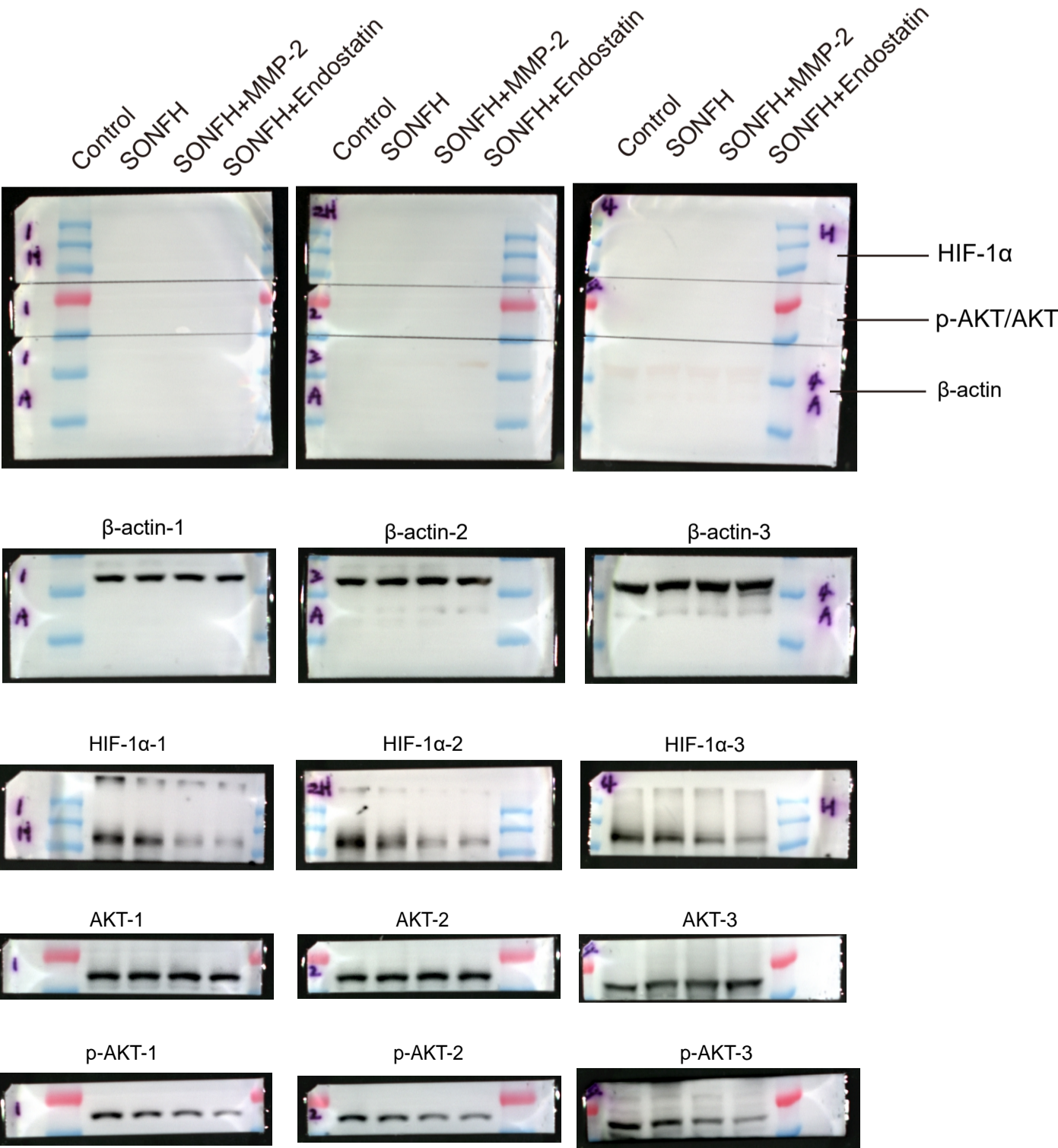

Supplement: S1 File — (PDF) [file pone.0346880.s003.pdf]
